# Prediction of virus–host interactions and identification of hot spot residues of DENV-2 and SH3 domain interactions

**DOI:** 10.1007/s00203-024-03892-x

**Published:** 2024-03-14

**Authors:** Mithila Banik, Keshav Raj Paudel, Rajib Majumder, Sobia Idrees

**Affiliations:** 1 Department of Bioinformatics and Biotechnology, Asian University for Women, Chattogram, Bangladesh; 2 Centre for Inflammation, Centenary Institute and the University of Technology Sydney, School of Life Sciences, Faculty of Science, Sydney, NSW Australia; 3 Applied Bioscience, Macquarie University, Sydney, NSW Australia

**Keywords:** Dengue, DENV-2, SH3 domain, Proline, Peptide-based vaccines

## Abstract

Dengue virus, particularly serotype 2 (DENV-2), poses a significant global health threat, and understanding the molecular basis of its interactions with host cell proteins is imperative for developing targeted therapeutic strategies. This study elucidated the interactions between proline-enriched motifs and Src homology 3 (SH3) domain. The SH3 domain is pivotal in mediating protein–protein interactions, particularly by recognizing and binding to proline-rich regions in partner proteins. Through a computational pipeline, we analyzed the interactions and binding modes of proline-enriched motifs with SH3 domains, identified new potential DENV-2 interactions with the SH3 domain, and revealed potential hot spot residues, underscoring their significance in the viral life cycle. This comprehensive analysis provides crucial insights into the molecular basis of DENV-2 infection, highlighting conserved and serotype-specific interactions. The identified hot spot residues offer potential targets for therapeutic intervention, laying the foundation for developing antiviral strategies against Dengue virus infection. These findings contribute to the broader understanding of viral–host interactions and provide a roadmap for future research on Dengue virus pathogenesis and treatment.

**Supplementary Information:**

The online version contains supplementary material available at 10.1007/s00203-024-03892-x.

## Introduction

Dengue Virus (DENV) has emerged as a significant global health threat, demanding an in-depth understanding of its intricate pathogenesis (Idrees and Ashfaq 2012; Chala and Hamde 2021; WHO 2021). As a prominent arthropod-transmitted viral disease causing substantial human morbidity and mortality, numerous studies have probed into Dengue’s pathogenesis (Bhatt et al. 2021). However, despite extensive research, the precise molecular mechanisms governing Dengue disease remain elusive, necessitating further exploration (Srikiatkhachorn 2009; Bhatt et al. 2013; Idrees and Ashfaq 2013; Kraemer et al. 2015; Guo et al. 2017). A hypothesis rooted in molecular mimicry suggests that certain Dengue-induced antibodies may cross-react with host proteins, and a study validated a direct association between the severity of secondary Dengue disease in humans and pre-existing anti-DENV antibody levels (Chuang et al. 2014). The interactions between Dengue and host proteins can be via Short Linear Motifs (SLiMs), that help viruses in hijacking the endosomal sorting complexes required for transport (ESCRT) (Idrees and Paudel 2023a, b; Idrees et al. 2023). Upon viral invasion, ESCRTs are recruited to the viral entry site by viral proteins with SLiMs (Ren and Hurley 2011; Venkatakrishnan et al. 2020; Idrees et al. 2023).

Previous studies have shown the significance of SH3 interactions in viral replication and pathogenicity. Notable instances include alphaviruses relying on NsP3-SH3 binding for RNA replication, HIV/SIV Nef proteins using an “R-clamp” strategy for kinase activation, HCV NS5A’s high-affinity interaction with the Fyn SH3 domain, and HIV/SIV Nef PxxP motifs promoting viral growth via SH3 binding (Saksela et al. 1995; Shelton and Harris 2008; Neuvonen et al. 2011; Ren and Hurley 2011; Zhao et al. 2021). Moreover, a study on mature DENV-2 virus has previously elucidated interactions within the E glycoprotein chains, partly mediated by the PXXP motif (Thomas and Endy 2011; Torres-Flores et al. 2022; Palanichamy Kala et al. 2023). As seen in related viruses and considering the therapeutic implications of similar motifs, further exploration of SH3 interactions in DENV-2 can provide insights into host–virus interactions and can aid in developing potential targets for antiviral strategies (Kaneko et al. 2008; Gould et al. 2009; Gadkari and Srinivasan 2010).

Therefore, in this study, we performed a network and structural approach to predict proline mediated interactions between DENV-2 proteins and the SH3 domain by investigating virus–host protein–protein interactions (PPIs) to understand DENV-2 molecular mechanisms and have identified hot spot residues involved in dengue infection which can be used as therapeutic targets.

## Methods

### Data retrieval and processing

Dengue virus (DENV) protein–protein interactions (PPIs) [n = 213] (Table S1) were downloaded from the Pathogen Host Interaction Search Tool (PHISTO) database (Durmus Tekir et al. 2013). These PPIs were used to predict new SLiM instances and domain–motif interactions (DMIs). UniProt IDs were mapped to their respective gene symbols, and viral protein sequence data for DENV-2 was retrieved from the UniProt database (Apweiler et al. 2004). Known structural domain data was downloaded from the 3did database (Mosca et al. 2014) that included SH3 resolved PDB structure (PDB ID: 5FW9).

### SLiM and DMI prediction

To predict new DMIs involving DENV-2, SLiM instances of known Eukaryotic Linear Motifs (ELM) were predicted using SLiMProb v2.5.1 (Edwards and Palopoli 2015). As SLiMs mostly reside in intrinsically disordered regions (IDRs) we used the disordered masking feature (IUPred score ≥ 0.2) to restrict the analysis to IDRs and refraining from any evolutionary filtering of results (Hagai et al. 2011). The predicted viral SLiMs were then used to predict DMIs using SLiMEnrich v1.5.1 (Idrees et al. 2018) through ELMc-Domain strategy (predicted viral SLiMs mapped to Pfam-domain-containing human partner proteins) available in SLiMEnrich (Idrees et al. 2018). SLiMEnrich works based on all ELM classes (e.g., LIG, DOC, DEG, MOD, CLV) by default. We restricted our analysis to proline-enriched motifs by downloading only LIG_SH3 motifs from ELM database and feeding it to SLiMEnrich, along with our PPI data. As the ELMc-Domain stringency is associated with a higher false discovery rate, we employed a computational structural approach to infer the likelihood of the identified interactions being real.

### Molecular docking analysis

3D structure of the SH3 domain belonging to SPTAN1 protein was downloaded from the 3DID database [PDB ID: 5FW9 (1.55 Å)]. Optimization of the structure was done by first removing H2O molecules and then doing 3D protonation to change the state to ionization level. This was done using “Dock Prep” function in UCSF Chimera software (Pettersen et al. 2004). Moreover, energy minimization was done using parameters such as force field: MMFF94X + Solvation, gradient 0.05, and chiral constraint: current geometry using UCSF Chimera software (Pettersen et al. 2004). This minimized structure was then used for docking through CABS-dock server. CABS-dock, based on the Computational Alanine Scanning (CABS) model, emphasizes the flexibility and dynamics of ligand–receptor interactions during docking, providing a more realistic portrayal of molecular dynamics. Particularly adept with unbound or partially flexible structures, CABS-dock accommodates significant conformational changes in binding partners, making it suitable for predicting binding modes in flexible proteins. Its computational efficiency, driven by an efficient sampling algorithm, enables the exploration of extensive conformational spaces within reasonable timeframes, proving advantageous for high-throughput or large-scale docking studies (Kurcinski et al. 2019). Following protein docking, the docked complexes underwent hydrogen bonding and buried surface interaction area analysis using PDBePISA (protein interfaces, surfaces, and assemblies service) server (Krissinel and Henrick 2007). Subsequently, FoldX (Schymkowitz et al. 2005) was employed to determine the binding energies within the peptide–protein complexes. FoldX offers a rapid and quantitative assessment of interaction significance in maintaining protein and protein complex stability. In addition, alanine scanning was performed using FoldX, a widely utilized method (Stein and Aloy 2008; London et al. 2010), particularly for predicting hot spot residues. Initially, the “RepairPDB” command rectified residues with unfavorable torsion angles, van der Waals clashes, or total energy issues. Subsequently, the “complex_alascan” command was employed to assess the contribution of each residue on the peptide’s interaction interface to the protein and peptide binding. This was achieved by estimating the binding free energy differences (ΔΔ*G*) following the mutation of the residue to Ala as shown.

$$\Delta \Delta G = \Delta G_{{{\text{mutant}}}} - \Delta G_{{{\text{wildtype}}}}$$

where Δ*G*_mutate_ and Δ*G*_wild_ are the binding free energies of the mutant complex and the wild-type complex, respectively.

### Conservation analysis

Polyprotein sequences of different serotypes of the Dengue virus were downloaded from the UniProt database (Apweiler et al. 2004). The UniProt Ids of sequences were serotype 1: P17763, serotype 2: P29991, serotype 3: Q6YMS4 and serotype 4: Q2YHF0. Sequences were aligned using the CLC workbench, and conservation analysis was done assessing the degree of similarity and preservation of specific sequences or structural motifs across different serotypes of Dengue virus. Conservation analysis helps researchers understand the evolutionary relationships, identify key functional elements, and inform the development of broad-spectrum treatments or vaccines.

## Results

### SLiM and DMI prediction

SLiMs were predicted using SLiMProb providing DENV-2 virus protein sequences involved in PPIs. SLiMs are mostly found in IDRs; therefore, the disordered masking feature was applied during SLiMProb run to predict new instances of known motifs that were in the IDRs. The predicted SLiMs along with PPIs were fed to SLiMEnrich v.1.5.1 to predict DMIs. SLiMEnrich works by mapping SLiMs to their interacting domain partners in the host proteins, giving the set of all possible DMIs. PPI data are then mapped to these DMIs to predict actual/real DMIs. A total of 23 DMIs were predicted involving 13 unique motifs instances from 3 ELM (i.e., LIG_SH3_1, LIG_SH3_2, LIG_SH3_3) in the DENV-2 polyprotein sequence (*E, NS1, NS3* and *NS5*) interacting with SH3 domain of three unique human protein partners (Table 1).

**Table 1:** Predicted DMIs involving SH3 motif instances

Motif	Start Pos	End Pos	Protein name	Pattern	SLiMProb sequence	Pfam domain	Human protein
LIG_SH3_3	406	412	E	…[PV]..P	XXXVVQP	PF00018	TP53BP2
LIG_SH3_3	417	423	E	…[PV]..P	YTIVITP	PF00018	TP53BP2
LIG_SH3_3	406	412	E	…[PV]..P	XXXVVQP	PF00018	SPTAN1
LIG_SH3_3	417	423	E	…[PV]..P	YTIVITP	PF00018	SPTAN1
LIG_SH3_1	808	814	NS1	[KRY]..P..P	KFQPESP	PF00018	SPTAN1
LIG_SH3_1	808	814	NS1	[KRY]..P..P	KFQPESP	PF00018	TP53BP2
LIG_SH3_2	811	816	NS1	P..P.[KR]	PESPSK	PF07653	FYB
LIG_SH3_2	811	816	NS1	P..P.[KR]	PESPSK	PF00018	SPTAN1
LIG_SH3_2	811	816	NS1	P..P.[KR]	PESPSK	PF00018	TP53BP2
LIG_SH3_3	808	814	NS1	…[PV]..P	KFQPESP	PF00018	TP53BP2
LIG_SH3_3	808	814	NS1	…[PV]..P	KFQPESP	PF00018	SPTAN1
LIG_SH3_3	1480	1486	NS3	…[PV]..P	WDVPSPP	PF00018	TP53BP2
LIG_SH3_3	1479	1485	NS3	…[PV]..P	LWDVPSP	PF00018	TP53BP2
LIG_SH3_3	1894	1900	NS3	…[PV]..P	AERVIDP	PF00018	TP53BP2
LIG_SH3_3	1480	1486	NS3	…[PV]..P	WDVPSPP	PF00018	SPTAN1
LIG_SH3_3	1479	1485	NS3	…[PV]..P	LWDVPSP	PF00018	SPTAN1
LIG_SH3_3	1894	1900	NS3	…[PV]..P	AERVIDP	PF00018	SPTAN1
LIG_SH3_3	3154	3160	NS5	…[PV]..P	DDCVVKP	PF00018	TP53BP2
LIG_SH3_3	2694	2700	NS5	…[PV]..P	GALVRNP	PF00018	TP53BP2
LIG_SH3_3	3068	3074	NS5	…[PV]..P	VVRVQRP	PF00018	TP53BP2
LIG_SH3_3	3154	3160	NS5	…[PV]..P	DDCVVKP	PF00018	SPTAN1
LIG_SH3_3	2694	2700	NS5	…[PV]..P	GALVRNP	PF00018	SPTAN1
LIG_SH3_3	3068	3074	NS5	…[PV]..P	VVRVQRP	PF00018	SPTAN1

### Docking and interaction analysis

The identified DMIs were then docked using the CABS-dock server (Kurcinski et al. 2019), where motif instances (peptides) identified in different DENV-2 viral proteins were docked with the native SH3 domain structure (Receptor, PDB ID: 5FW9) and looked at close contact possible interactors, i.e., pairs of peptide/receptor residues closer than 4.0 Å in the selected complex. To see, if there were actual interactions between any of these residues, we conducted hydrogen bond analysis using PDBePISA (Krissinel and Henrick 2007) server by uploading our newly docked domain–motif complexes and identified hydrogen bond between only different residues of SH3 domain and DENV-2 proteins, suggesting a possible interaction between both residues. In simpler terms, we found specific connections (like hydrogen bonds and salt bridges) between different DENV-2 proteins, e.g., E, NS1, NS3, NS5, and SH3 receptor providing important details about how these proteins work and remain stable during a DENV-2 infection (Table 2).

**Table 2:** Interacting residues between DENV-2 peptide and SH3 domain

Viral protein	Peptide	Receptor residue	DENV-2 peptide residue	Interaction dist. (Å)	Interaction type
Envelope	417–423	SER 14 [OG]	THR 6 [OG1]	3.81649	H bond
NS1	808–814	LYS 13[ N]	SER 6[ OG]	2.74	H bond
		GLN 11[ N]	PRO 7[ O]	2.62	H bond
		LYS 13[ O]	GLU 5[ N]	3.90	H bond
		TYR 10[ OH]	SER 6[ N]	3.78	H bond
	811–816	GLN 11[ N]	PRO 1[ O]	3.54	H bond
		TYR 10[ OH]	SER 3[ OG]	2.62	H bond
		ASP 9[ O]	PRO 1[ N]	3.61	H bond
		GLU 17[ OE1]	SER 3[ OG]	3.72	H bond
		SER 14[ OG]	LYS 6[ N]	2.90	H bond
NS3	1479–1485	TYR 10[ OH]	TRP 2[ O]	3.68	H bond
		TYR 10[ OH]	ASP 3[ OD1]	3.49	H bond
		SER 14[ N]	ASP 3[ OD2]	2.86	H bond
	1480–1486	ASN 30[ ND2]	TRP 1[ O]	3.68	H bond
		SER 14[ OG]	B:SER 5[ OG]	3.68	H bond
		SER 14[ OG]	PRO 6[ O]	3.86	H bond
		THR 32[ OG1]	TRP 1[ NE1]	3.52	H bond
	1894–1900	LYS 13[ NZ]	ALA 1[ O]	3.09	H bond
		ASN 30[ ND2]	ASP 6[ OD1]	3.58	H bond
NS5	2694–2700	SER 14[ OG]	VAL 4[ O]	3.89	H bond
		ASP 35[ OD2]	ARG 5[ NH1]	2.66	H bond
					Salt bridge
	3068–3074	TYR 8[ OH]	VAL 1[ N]	3.71	H bond
		TYR 10[ OH]	VAL 4[ N]	3.07	H bond
	3154–3160	ARG 16[ NH2]	ASP 1[ OD2]	3.38	H bond
					Salt bridge
		SER 14[ N]	LYS 6[ O]	2.74	H bond
		TYR 10[ OH]	PRO 7[ OXT]	2.60	H bond
		ASN 33[ OD1]	ASP 1[ N]	3.28	H bond
		GLU 17[ OE2]	LYS 6[ N]	2.67	H bond
		TYR 10[ OH]	PRO 7[ N]	3.00	H bond
		GLU 17[ OE2]	PRO 7[ N]	3.80	H bond

### Energy calculations and alanine scanning

**Fig. 1:**
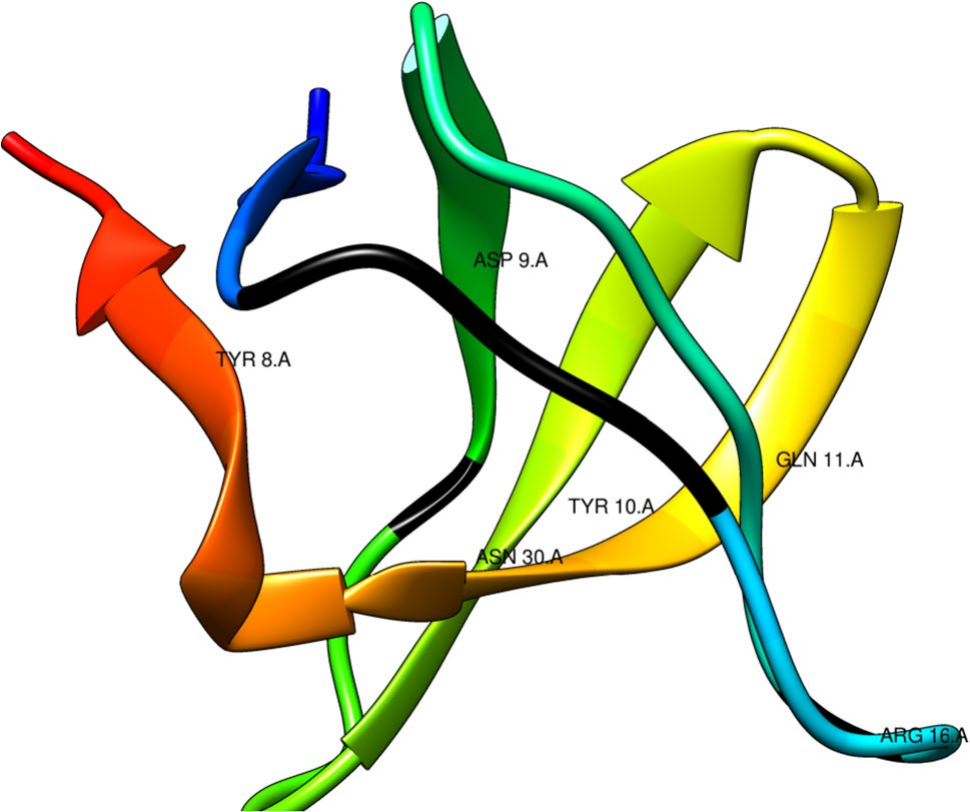
Hot spot residues of SH3 domain identified to be interacting with DENV-2 proteins

The FoldX package was utilized to compute the binding energy (Δ*G*) of the peptide–domain complex. In addition, alanine scanning mutagenesis, a widely adopted technique involving the systematic replacement of selected residues with alanine, was conducted using FoldX to identify crucial peptide residues for binding. This method systematically replaces selected residues in a target protein with alanine through site-directed mutagenesis. Alanine scanning facilitates the identification of specific amino acid residues crucial for peptide binding, as alanine substitutions eliminate side-chain interactions without affecting the main-chain conformation or introducing steric or electrostatic effects, preserving the native protein structure. Six hot spot residues were identified, including TYR 8, ASP 9, ASN 30, GLN 11, ARG 16, and TYR 10. A hot spot is defined as a residue exhibiting a ΔΔ*G* ≥ 1.0 kcal/mol upon mutation to alanine, following the criteria established by Kortemme et al. (2004) and Grosdidier and Fernandez-Recio (2008). These residues exhibited various interaction types, with identified DENV-2 peptides forming strong hydrogen bond interactions. In addition, ARG 16 engaged in both hydrogen bond interactions and salt bridges. The reduction in peptide binding upon substituting an essential amino acid serves as a relative measure of its importance in the context of the substitution (Table 3, Fig. 1).

**Table 3:** Hot spot residues of SH3 domain involved in interactions with DENV-2 SH3 motifs

Hot spot residue	ΔΔ*G* (kcal/mol)	Interaction type
TYR 8	1.77917	H bond
ASP 9	1.22976	H bond
TYR 10	4.53748	H bond
GLN 11	1.20148	H bond
ARG 16	1.10809	H bond
		Salt bridge
ASN 30	2.21985	H bond

### Conservation analysis of SH3 motifs

**Fig. 2:**
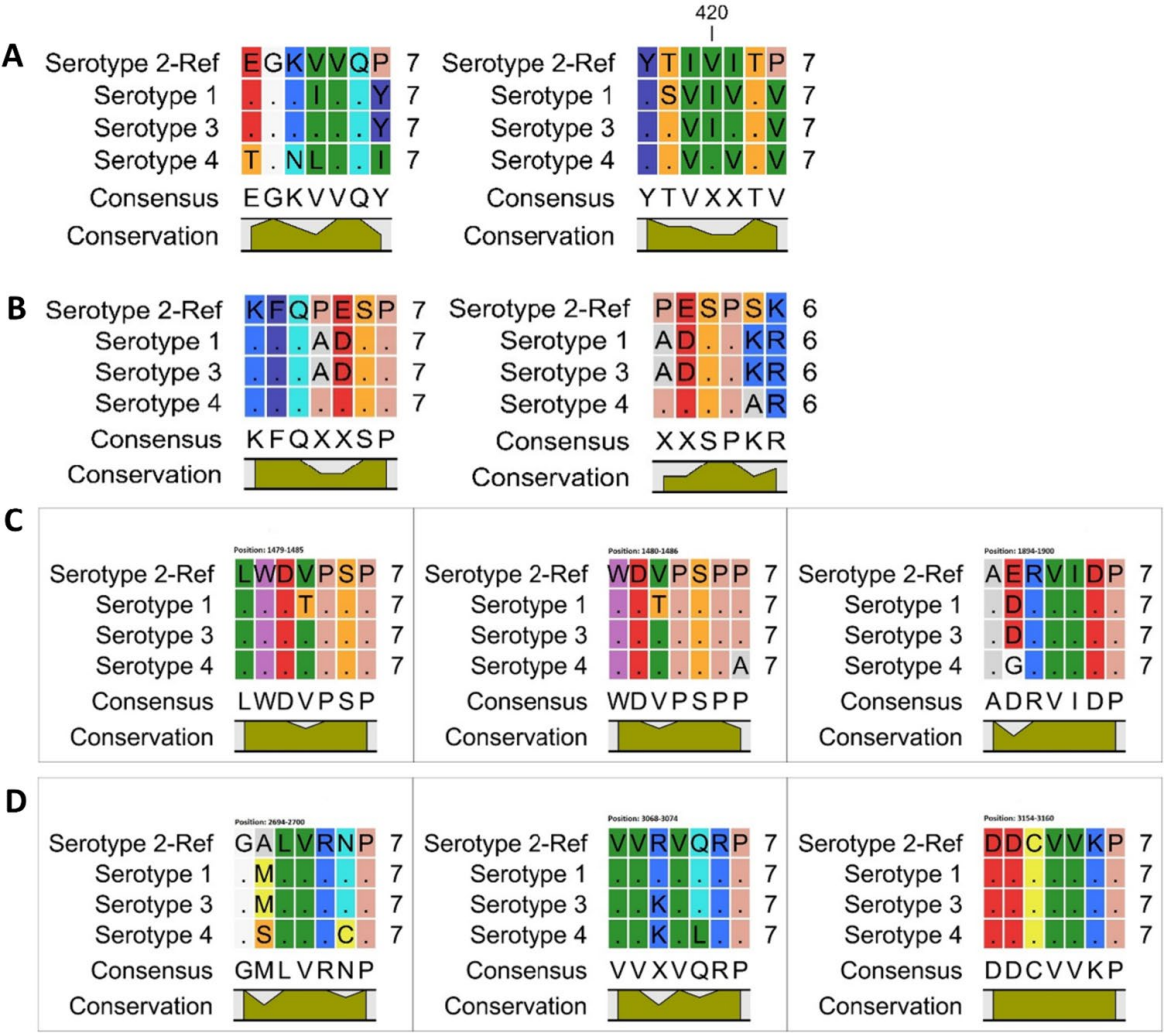
Conservation analysis of identified proline-enriched peptides in different Dengue serotypes, **A** envelope protein peptides, **B** NS1 protein peptides, **C** NS3 protein peptides, **D** NS5 protein peptides. Conservation is shown at the bottom in green. Dot (.) shows identical/conserved residues. Alanine (A) residues represented as grey, arginine (R) and lysine (K) as light blue, asparagine (D) and glutamate (E) as red, cysteine (C) and methionine (M) as yellow, glycine (G) as white, histidine (H) as purple, isoleucine (I), leucine (L), and valine (V) as green. Phenylalanine (F), tyrosine (T) as dark blue, proline (P) as flesh, serine (S) and threonine (T) as orange, tryptophan (W) as orchid (color figure online)

Polyprotein sequences representing diverse serotypes of the Dengue virus were sourced from the UniProt database (Apweiler et al. 2004). The UniProt identifiers for these sequences are as follows: P17763 for serotype 1, P29991 for serotype 2, Q6YMS4 for serotype 3, and Q2YHF0 for serotype 4. Employing the CLC Workbench, we aligned these sequences, keeping DENV-2 as reference and conducted a conservation analysis to evaluate the degree of similarity and preservation of specific sequences or structural motifs across the distinct Dengue serotypes. The envelope protein revealed residues, such as GLN at position 411 and THR at position 418, with conserved interactions, emphasizing their pivotal role in binding. Conversely, ILE at position 419 displayed non-conservation, highlighting the context-specific nature of certain interactions. In addition, VAL at position 420 exhibited specificity for serotype 4, adding a layer of complexity to the binding environment. In the NS1 protein, residues like GLN at position 810 and GLU at position 812 displayed strong conserved interactions, underscoring their significance in the viral life cycle. Notably, the identified hot spot residues in NS1, including SER at position 813 and PRO at position 814, further contribute to our understanding of the intricate molecular mechanisms governing DENV-2 infection. Within the NS3 protein, the conserved interactions of TRP at position 1480, SER at position 1484, and PRO at position 1485, contrasted with the non-conservation of GLU at position 1895, showcase the diverse landscape of interactions within the viral polyprotein. Meanwhile, ARG at position 1896 underscores the strategic conservation of certain residues critical for binding. The NS5 protein revealed a network of conserved interactions, with residues such as VAL at positions 2697, 3068, 3069, 3071, and others, indicating their essential roles in viral–host interactions. Notably, ARG at position 3070 exhibited serotype specificity, adding a layer of selectivity to the binding interactions. The diverse array of interactions identified in this study contributes to our understanding of the molecular basis of DENV-2 infection and provides a foundation for the design of targeted therapeutic interventions. These findings pave the way for further exploration into the specific roles of these residues in viral entry, replication, and immune evasion. In addition, the conservation patterns highlighted in this study may have implications for the development of broad-spectrum antiviral strategies targeting shared elements across different serotypes (Table 4, Fig. 2).

**Table 4:** DENV-2 interacting residues with SH3 domain and their conservation with other serotypes (DENV-1-4)

Peptide interacting residue	Position in DENV-2 polyprotein	Protein	Conservation with other serotypes
GLN	411	Envelope	Yes
THR	418	Envelope	Serotype 3, 4
ILE	419	Envelope	No
VAL	420	Envelope	Serotype 4
THR	422	Envelope	Yes
GLN	810	NS1	Yes
GLU	812	NS1	Serotype 4
SER	813	NS1	Yes
PRO	814	NS1	Yes
SER	815	NS1	No
TRP	1480	NS3	Yes
SER	1484	NS3	Yes
PRO	1485	NS3	Yes
GLU	1895	NS3	No
ARG	1896	NS3	Yes
VAL	2697	NS5	Yes
ARG	2698	NS5	Yes
VAL	3068	NS5	Yes
VAL	3069	NS5	Yes
ARG	3070	NS5	Serotype 1
VAL	3071	NS5	Yes
ASP	3154	NS5	Yes
ASP	3155	NS5	Yes
VAL	3157	NS5	Yes
LYS	3159	NS5	Yes

## Discussion

Dengue infection has become a global threat to human health. Dengue virus has four serotypes, which hinders development of a successful vaccine (Idrees and Ashfaq 2012, 2013). The comprehensive analysis of Dengue Virus Serotype 2 (DENV-2) molecular interactions presented in this study sheds light on the viral–host protein-protein interactions (vhPPIs), providing valuable insights into potential therapeutic targets. Our exploration encompassed docking studies, interaction analyses, energy calculations, and alanine scanning mutagenesis, identifying specific residues critical for the stability and functionality of viral proteins during infection. The SH3 domain interactions with proline-rich motifs have been extensively studied in various viruses. SH3 domains are modular protein–protein interaction domains that specifically recognize and bind to proline-rich motifs, often found in viral and cellular proteins (Kaneko et al. 2008; Neuvonen et al. 2011; Zhao et al. 2021; Tossavainen et al. 2022). The presence of proline-rich motifs in DENV-2 proteins, such as those identified in this study, suggests a potential mechanism for viral proteins to exploit host cell machinery through SH3 domain interactions.

In this study, the application of SLiMProb, specifically considering IDRs through the disordered masking feature, yielded a comprehensive prediction of SLiMs within the DENV-2 polyprotein. SLiMs are often located in IDRs, and their prediction within these regions provides insights into potential interaction interfaces crucial for the virus life cycle. In this study, we focused on three proline-enriched motifs (LIG_SH3_1, LIG_SH3_2, LIG_SH3_3) within IDRs. The subsequent integration of predicted SLiMs into SLiMEnrich facilitated the prediction of DMIs, mapping SLiMs to their interacting domain partners in host proteins. This step is pivotal for understanding the broader context of vhPPIs, as it moves beyond individual motifs to predict the specific domains within host proteins that may be targeted by the virus. The focus on SH3 domains, known for their involvement in various cellular processes including signal transduction, emphasizes the strategic selection of host cellular components by the virus. The results showcased a total of 23 predicted DMIs involving 13 unique motif instances positioned in DENV-2 polyproteins, including E, NS1, NS3, and NS5, engaging with two unique domains in three distinct human protein partners (TP53BP2, SPTAN1, FYB). The variety of motifs and host protein partners underscore the intricacies of DENV-2 interactions, demonstrating the virus’s ability to target multiple cellular pathways. Molecular docking analyses then further assessed the predicted DMIs, providing a detailed view of specific peptide–residue interactions within the DENV-2 polyprotein and host SH3 domains. The strength and likeliness of interactions were further confirmed using hydrogen bond analysis. As we know, hydrogen bonds between specific amino acid residues in the protein and functional groups on the ligand contribute to the specificity of these interactions. This is crucial for processes such as enzyme–substrate binding or receptor–ligand interactions (Morrow and Zhang 2012). The incorporation of hot spots in subsequent analyses enhances our understanding of the contribution of specific residues to the overall binding free energy. The computation of binding energy (ΔΔ*G*) using the FoldX package identified six hot spot residues crucial for binding. These residues exhibited various interaction types, such as hydrogen bonds and salt bridges, emphasizing their significance in maintaining the stability of vhPPIs. The concept of hot spots is instrumental in identifying key residues contributing significantly to the binding free energy. The distribution of energy across PPIs is not uniform, and a subset of residues, termed “hot spots,” significantly contributes to binding free energy (Clackson and Wells 1995; Keskin et al. 2005). Clackson and Wells’ pioneering study established the concept of hot spots, highlighting residues whose mutation to alanine results in a significant decrease in binding free energy (ΔΔ*G*) (Clackson and Wells 1995). Hot spots occupy a fraction of the larger interface area and exhibit structural conservation (Bogan and Thorn 1998). Extensive research on protein–protein interfaces has identified specific hot spots. These regions, comprising only a small fraction of interfacial residues, play a crucial role in determining binding affinity and specificity (DeLano 2002). Structural conservation of hot spots is crucial in understanding the cooperative nature of these residues. Hot spots have been considered in drug design, serving as attractive targets for small molecule inhibitors to disrupt unwanted PPIs. The conservation of hot spots and their correlating binding affinity make them promising targets for drug development, offering avenues for small molecule inhibition of specific interactions. The consideration of hot spots in the design of small molecule inhibitors presents two key avenues for drug development. First, the presence of hot spots aids in predicting the binding site, guiding the docking, and screening of potential ligands. Second, the relatively less flexible nature of hot spots can be exploited in rigid docking approaches, improving the accuracy of protein docking by considering dominant conformational states obtained from molecular dynamics simulations (Gonzalez-Ruiz and Gohlke 2006; Morrow and Zhang 2012). The binding energy (ΔΔ*G*) of the peptide–domain complexes was computed using the FoldX package. Furthermore, a conservation analysis was conducted across different serotypes of Dengue virus polyproteins (DENV 1-4), revealing the preservation of specific residues involved in interactions. The conservation patterns highlighted the significance of certain residues in the context of viral–host interactions, offering potential targets for therapeutic interventions. In general, the incorporation of hot spots in the analysis of DENV-2 molecular interactions enhances our understanding of the energetics and structural aspects of vhPPIs. This knowledge holds promise for the design of targeted therapeutic interventions aimed at disrupting key PPIs essential for DENV infection.

## Conclusion

This study investigated interactions between the proline-enriched motif and SH3 domains in Dengue virus serotype 2 (DENV-2). Through a comprehensive analysis, we have uncovered significant insights into the role of proline-enriched motifs in mediating crucial interactions with SH3 domains, shedding light on DENV-2 interactions. Our findings suggest that the proline-enriched motifs play a pivotal role in facilitating interactions with SH3 domains, contributing to the network of protein–protein interactions that govern viral replication and host cell manipulation. Moreover, the identification and characterization of specific proline-enriched motifs involved in SH3 interactions offer potential targets for antiviral drug development. By disrupting these critical interactions, we may be able to impede the progression of DENV-2 infection and mitigate its impact on human health.

### Supplementary Information

Below is the link to the electronic supplementary material.

Supplementary file 1 (CSV 3 kb)

## Data Availability

The PPI data used in this study are available as supplementary file.
